# TRAJECTORIES OF K-12 SCHOOLS IN THE 1930S-1960S AND COGNITIVE AGING: VARIATION BY RACIAL COMPOSITION

**DOI:** 10.1093/geroni/igac059.203

**Published:** 2022-12-20

**Authors:** Wenshan Yu, Jacqui Smith

**Affiliations:** University of Michigan, Ann Arbor, Michigan, United States; University of Michigan, Ann Arbor, Michigan, United States

## Abstract

Besides information about the highest degree, little information about early-life education is available in population surveys. We identified K-12 education trajectories among older adults in the Health and Retirement Study and examined their association with cognitive function. Drawing on 2017 Life History Mail Survey (n = 4,325), we used sequence analysis to determine and classify trajectories of school majority race. We identified five clusters: 1) mostly White schools (n = 3,027), 2) mostly Black schools (n = 673), 3) mostly Hispanic schools (n = 499), 4) partly missing on majority race (n = 267), and 5) mostly Mixed-race schools (n =113). Adding the cluster variable significantly improved the model regressing cognitive function on race, gender, number of years in schools, birth cohort, number of years since starting schools, and school locations (F=12.066, p<0.001). Future research should study heterogeneity of K-12 education using information other than the highest degree.

